# Various Types of Light Guides for Use in Lossy Mode Resonance-Based Sensors

**DOI:** 10.3390/s23136049

**Published:** 2023-06-30

**Authors:** Dmitriy P. Sudas, Viktor A. Jitov, Petr I. Kuznetsov

**Affiliations:** 1 Kotel’nikov Institute of Radioengineering and Electronics of the Russian Academy of Sciences (Fryazino Branch), sq. Vvedenskogo 1, Fryazino, Moscow 141190, Russia; 2 World-Class Research Center, Peter the Great St. Petersburg Polytechnical University, Polytechnicheskaya ul.29, St. Petersburg 195251, Russia

**Keywords:** lossy mode resonance, fiber chemosensor, figure of merit, ZnTe, MOCVD

## Abstract

A comparative study of figure-of-merit fiber sensors of the mass concentration of NaCl solutions based on single-mode and multi-mode fibers was carried out. Lossy mode resonance is realized on chemically thinned sections of optical fibers to various diameters (from 26 to 100 μm) coated with ZnTe. Thin-film coatings were applied using the method of metalorganic chemical vapor deposition (MOCVD). Samples of single-mode and multi-mode fiber sensors were created in such a way that the depth and spectral position of resonances in aqueous NaCl solutions coincided. Sensors implemented on a single-mode fiber have a higher sensitivity (5930 nm/refractive index unit (RIU)) compared to those on a multi-mode fiber (4860 nm/RIU) and a smaller half-width of the resonance in the transmission spectrum. According to the results of experiments, figure-of-merit sensors are in the range of refractive indices of 1.33–1.35 for: multi-mode fiber—25 RIU^−1^, single-mode fiber—75 RIU^−1^. The sensitivity of the resulting sensors depends on the surface roughness of the ZnTe coating. The roughness of films synthesized on a single-mode fiber is four times higher than this parameter for a coating on a multi-mode fiber. For the first time, in the transmission spectrum during the synthesis of a thin-film coating on a multi-mode fiber, the possibility of separating the first nine orders of resonances into electric and magnetic transverse components has been demonstrated. The characteristics of sensors with the operating wavelength range in the visible (500–750 nm) and infrared (1350–1550 nm) regions of the spectrum are compared. The characteristics of multi-mode lossy mode resonance sensors are demonstrated, which make them more promising for use in applied devices than for laboratory research.

## 1. Introduction

Currently, monitoring systems based on the optical fiber are actively used in many fields of science and technology. The high chemical resistance of the silica surface of the optical fibers allows them to be used in the most dangerous and aggressive environmental conditions. In particular, fiber sensors make it possible to determine the pH [1,2,3], number of antibodies [4,5,6], relative humidity [7,8,9], temperature [10,11,12], gas composition [13,14,15], and presence of volatile organic compounds [16,17,18]. All of the above parameters are estimated from the change in the refractive index near the sensitive surface of the fiber sensors; therefore, such sensors can be attributed to the class of devices—refractometers. The sensitive part of such devices is the region of the light guide in which the interaction of the light propagating in the core with the environment takes place. The enhancement of this interaction is implemented by methods of changing the geometry of the optical fiber in the local area [4,7,19,20,21], applying thin-film coatings [1,22,23,24], or combining the first and second [1,4,7,10,13,14,15]. In addition, microstructured fibers [25], fibers with two claddings [26], or those without a cladding [27] are also used to create sensors.

The most common subspecies of fiber refractometers is based on the phenomenon of lossy mode resonance (LMR)—the effect of transferring propagating energy from the core to an optically transparent cladding and, further, to the environment [28,29,30]. The interference between the fundamental mode and the coverage modes appears as a dip in the transmission spectrum of the optical path. The position of the resonance maximum—the point with the minimum transmission value in the spectrum of the optical path, depends on the refractive indices of the core and the cladding of the optical fiber, as well as the same parameter of the coating and the environment. In addition, the real part of the dielectric constant of the coating material must exceed its imaginary part in absolute value. In this case, it must be positive and greater than the same parameter for the waveguide and the environment [29]. Moreover, the real part of the dielectric constant of the coating film must be positive. The value of the shift of the position of the resonance in the transmission spectrum when the sensor is placed in a specific medium determines its sensitivity, and the greater it is, the greater the refractive index of the coating used is [31].

The coating material can be different compounds [32,33,34], and strictly defined compounds are selected for specific tasks. For example, Indium-Tin oxide (ITO) coatings [35,36], due to the large number of dangling bonds on the surface and their porous structure, are well suited for working with gaseous media. Tin oxide (SnO_2_) [34,37,38] has an extremely high chemical stability and is stable even in concentrated acids. However, due to the high hardness of the material and the large difference in thermal expansion coefficients between tin oxide and silica fiber, finished sensors have a low mechanical strength. Silicon nitride (Si_3_N_4_) is used in biosensors [39], and due to its antibacterial properties and excellent biogenesis [40,41], this material has also recently been actively used in medicine. However, surface cracking often occurs [42] due to the high internal stresses that occur during synthesis, which limits the use of this coating in final devices. The hardness difference factor imposes serious restrictions on the possible configurations of sensor structures, especially if their widespread use is implied. Zinc telluride (ZnTe) films are also subject to cracking in some cases, but they are of interest for research purposes due to the high refractive index in the spectral region from 0.6 to 2 microns, as well as the ease of their application and, if necessary, their removal from the silica surface.

To create sensors based on the LMR phenomenon, standard telecommunication optical fibers, both multi-mode (MMF) and single-mode (SMF), are usually used. Sensors based on a multi-mode fiber are mechanically more robust due to the wide core compared to single-mode fibers. This fact leads to the fact that the diameter of the fiber section at the point of contact between the optical energy in the core and the coating is larger than the same parameter for implementation on a single-mode fiber. However, in this case, a large number of interacting modes leads to the fact that the resonance has a significant width. This reduces the accuracy of determining the resonance maximum and worsens the detection limit. To characterize sensors, a parameter such as figure of merit (FOM) is important, which is the ratio of the sensor sensitivity to the half-width of the resonance in the transmission spectrum. Therefore, sensors based on multi-mode fibers, which have wider resonances, potentially have a lower FOM compared to similar devices on single-mode fibers.

In this study, for the first time, a comparison of the figure-of-merit LMR-sensors based on single-mode and multi-mode fibers was carried out. For this purpose, zinc telluride coatings on a series of chemically thinned single-mode and multi-mode silica fibers were synthesized by metal-organic chemical vapor deposition (MOCVD). First, the method we used makes it possible to deposit uniform thin-film coatings on the complex cylindrical surface of an optical fiber. Alternative methods involve fiber rotation, but when working with chemically thinned fibers, mechanical movement is not desirable. In addition, when using the MOCVD technique for the synthesis of zinc telluride, it is quite easy to obtain the desired stoichiometry. The resulting structures were studied as sensors for the concentration of aqueous solutions of salt (NaCl). The excellent optical quality of the surface and the high thickness uniformity of the ZnTe coatings made it possible to fix many orders of LMRs on a multi-mode fiber. A comparison is made of the sensitivities of sensors based on multi-mode fibers in the visible and infrared regions (IR) of the spectrum. In addition, the sensitivities of single-mode and multi-mode salt concentration sensors in the near-IR wavelength range were compared.

## 2. Materials and Methods

Standard single-mode SMF-28 fibers with a core diameter of about 8 microns, as well as multi-mode fibers of our own production with a diameter of 95 microns, were chemically thinned using a low-toxic polishing etchant based on NH_4_F and (NH_4_)_2_SO_4_ (Merck KGaA, Darmstadt, Germany). After the removal of the protective polymer, short sections of fibers about 2.0 mm long were successively flooded with an etchant and washed with distilled water, recording the diameter and quality of the surface on an optical microscope at each stage of etching. The thinning of the fiber was stopped when the diameter required for a particular experiment was reached. The process of the formation of thinned sections of optical fibers for use in sensorics is described in detail in [43,44].

**Figure 1:**
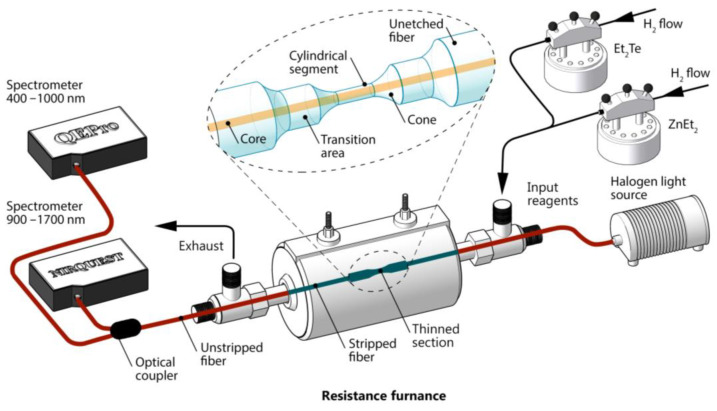
Scheme of the deposition of a ZnTe-coating on a silica fiber and controlling the optical transmission. The inset shows a diagram of a thinned fiber section.

The prepared fiber was placed in a tubular quartz reactor with an inner diameter of 5.5 mm and sealed at both ends. The center of the thinned fiber section was aligned with the center of the cylindrical resistive furnace. The MOCVD coating process was carried out at an atmospheric pressure of hydrogen with a dew point below −90 °C. The temperature inside the reactor in the area of the thinned section of the fiber was 270 °C. Diethylzinc (ZnEt_2_) and diethyltelluride (Et_2_Te) were used as initial reagents. They were thermostated in stainless steel containers at 15 and 20 °C, respectively. The electronic gas flow controllers and thermal concentration sensors used in the work made it possible to precisely change the ratio of the partial pressures of the reagents in the precipitation processes. In all processes, the hydrogen flow through the bubbler with ZnEt_2_ was 7.5 cm^3^/min, while the hydrogen flow through the bubbler with Et_2_Te was varied to achieve the desired ratio of reactants. The linear velocity of the vapor–gas mixture in the reactor varied from 15 to 25 cm/s. During the deposition process, the optical transmission of the fiber was in situ controlled in the wavelength range of 400–1700 nm using two spectrometers. Therefore, the operating range of the sensors obtained in the work will be the range of the spectrometers used. A schematic of the coating process with optical transmission control is shown in Figure 1. The light source was an HL-2000 OceanOptics halogen lamp. To control the transmission spectrum, NIRQuest-512 and QEPro spectrometers, also from OceanOptics Inc. (Rochester, NY, USA), were used. 

After finishing the deposition process, the coated fiber was removed from the reactor and kept in air at room temperature for 12 h before testing. Within a few hours after deposition, the final temperature stabilization of the surface occurs. Moisture from the environment penetrates into the pores of the coating material; in addition, oxidation processes end during exposure to air. The fiber samples were carefully examined under a microscope for the optical quality of the surface, the presence of cracks and other defects on it. Cracking of the coating occurs only in the case of incorrectly selected conditions for synthesis and cooling. If the coating is obtained without cracks, then it has a mechanical strength no less than that of the optical fiber on which the coating is applied. For a number of samples, surface images were taken at a high magnification on a scanning electron microscope (SEM) with a tungsten thermionic cathode JSM-6480LV (Jeol Ltd., Tokyo, Japan). Sensor samples selected for sensitivity measurements were filled several times with distilled water until the resonance position stabilized. The sensors were tested in aqueous solutions of NaCl, the mass concentration of which varied from 0 to 9 percent. In a number of growth experiments, to determine the optical constants of the coatings by ellipsometry, thin quartz plates were placed under a thinned fiber section. The values of the refractive index and the extinction coefficient were obtained on an ellipsometer according to the procedure described in detail in [45]. Information about the surface roughness was obtained by AFM using an NT-MDT INTEGRA Prima scanning probe microscope operating in a semi-contact mode. Silicon cantilevers NT-MDT of the Golden series NSG01 were used as probes.

For the greater repeatability of the results, the same fiber section with a thinned section was used in some measurement cycles. This was possible due to the ease of the removal of the studied or tested coating in a solution of a mixture of concentrated hydrochloric acid (HCl) with hydrogen peroxide (H_2_O_2_). The quartz reactor was cleaned with the same solution after each deposition process. 

## 3. Results and Discussion

**Figure 2:**
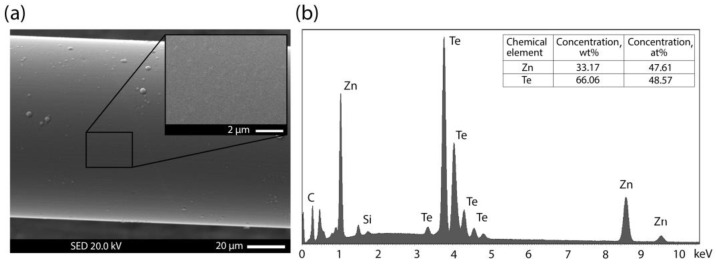
Characterization of the ZnTe coating with a thickness of 420 nm deposited at a temperature of 270 °C. (**a**) SEM images of the side surfaces of an optical fiber coated with a synthesized film; the inset shows the surface at a higher magnification. (**b**) EDX spectrum of the presented area. In addition to the Zn and Te shown in the table, there are also elements from the surface of the optical fiber, which account for less than 1 wt%.

In order to correctly compare the influence of the type of fiber network guide on the characteristics of a refractometer, it was necessary to solve several interrelated problems: first, the selection of the necessary coating material and the conditions for its synthesis; second, the choice of the operating wavelength range. In addition, it is necessary to choose the geometric parameters of the optical fiber thinned segment. Zinc telluride, chosen as the coating material for the formation of the lossy mode resonance, is convenient not only because of the ease of its removal from the silica surface of the optical fiber but also because of the ease of obtaining films of the stoichiometric composition of ZnTe. Some of the deposited sensor coatings were studied using an electron microscope; the elemental composition of the obtained film was checked by X-ray energy dispersive analysis (EDX). Figure 2 shows the results of such a study for one of the samples based on a multi-mode fiber. 

**Figure 3:**
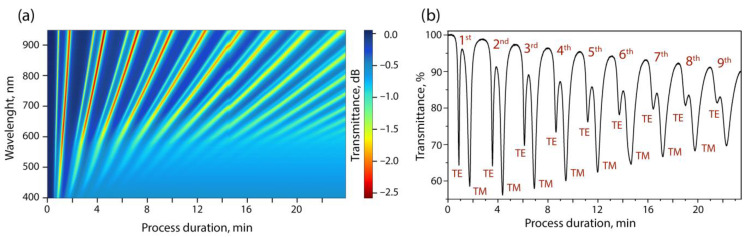
(**a**) Spectral sweep of resonances in the process of the deposition of a ZnTe coating on the surface of a multi-mode fiber; (**b**) cross-section at a wavelength of 900 nm.

In situ monitoring of the transmission spectrum of a fiber path with a thinned section made it possible to observe the emerging resonances and their movement along the wavelength in the process of increasing the coating thickness. As we have shown earlier [46], it is possible to estimate not only the thickness but also the dispersion of the refractive index of the coating being grown from the spectral sweeps from the deposition time along the position and depth of the resonances. However, this requires that at least the first three orders of resonances be separated into transverse electrical (TE) and transverse magnetical (TM) LMRs components. The considered modes are the result of the interaction of the HE_1,1_ mode of the optical fiber with the mode supported by the coating. In the case of interaction with the TE and TM components, the thin-film modes in the transmission spectrum of the dielectric waveguide are observed to be TE- and TM-LMR, respectively. On single-mode fibers, this separation is achievable [38,44] due to the small resonance width even without the use of polarizers. Usually, the large width of the resonances that appear in multi-mode fibers does not allow them to be divided into components already of the second order. However, this turned out to be possible when depositing ZnTe coatings on a thinned multi-mode fiber by the MOCVD method. Figure 3 shows the spectral sweep of one of the processes carried out on an MMF with a thinned section diameter of 80 microns.

Based on the Rayleigh criterion, it is possible to divide the first nine resonance orders into mode components in the transmission spectrum. According to our data, such a result is demonstrated in the literature for the first time. The separation was achieved due to the low value of the imaginary part of the complex refractive index of the coating, the high thickness uniformity, and the optical quality of the surface of the deposited ZnTe film. Due to this, a multi-mode fiber, like a single-mode fiber, can be used to calculate the optical constants of thin-film coatings. Note that the experimental process of deposition on a multi-mode fiber is much easier to perform due to its greater mechanical strength. It is known that, in dielectric waveguides with a semiconductor coating, the minimum optical transmission (resonance) of light propagating through it is observed when the cutoff conditions for the mode supported by a thin semiconductor layer are met [47]. 

Consider an asymmetric three-layer dielectric waveguide in which an isotropic dielectric of thickness d, with a refractive index $n_{1}$ is located between two semi-infinite layers with lower refractive indices $n_{2}$ and $n_{3}$ ($n_{1}$ > $n_{2}$ ≥ $n_{3}$). Under $n_{2}$ and $n_{3}$ are taken the refractive indices of silica glass and the environment, respectively. The coating thickness criteria for the cutoff at wavelength *λ* for the TE and TM modes of the *N*-th order in such a structure will be [31]:

(1) $$d_{N_{LMR}TE}=\frac{\lambda }{2\pi {\left(n_{1}^{2}-n_{2}^{2}\right)}^{\frac{1}{2}}}\left[arctan\left[{\left(\frac{n_{2}^{2}-n_{3}^{2}}{n_{1}^{2}-n_{2}^{2}}\right)}^{\frac{1}{2}}\right]+N\pi \right]$$

(2) $$d_{N_{LMR}TM}=\frac{\lambda }{2\pi {\left(n_{1}^{2}-n_{2}^{2}\right)}^{\frac{1}{2}}}\left[arctan\left[{{\left(\frac{n_{1}}{n_{3}}\right)}^{2}\left(\frac{n_{2}^{2}-n_{3}^{2}}{n_{1}^{2}-n_{2}^{2}}\right)}^{\frac{1}{2}}\right]+N\pi \right]$$

where N is the order of resonance minus one (N = $N_{LMR}-1$), in which case the first pair of LMRs ($N_{LMR}$ = 1) is reached at N = 0. In the obtained pairs of dips, the first resonance is due to the interaction of the fundamental mode with the TE coating component, since the necessary cutoff conditions for this component are met at a shorter wavelength than for the TM component. Therefore, by knowing the type of resonance and the refractive indices fiber and environments, it is possible to calculate the necessary film thickness to observe the resonance in the selected region [31]. In this case, it is necessary to first estimate the value of the refractive index of the grown material. 

**Figure 4:**
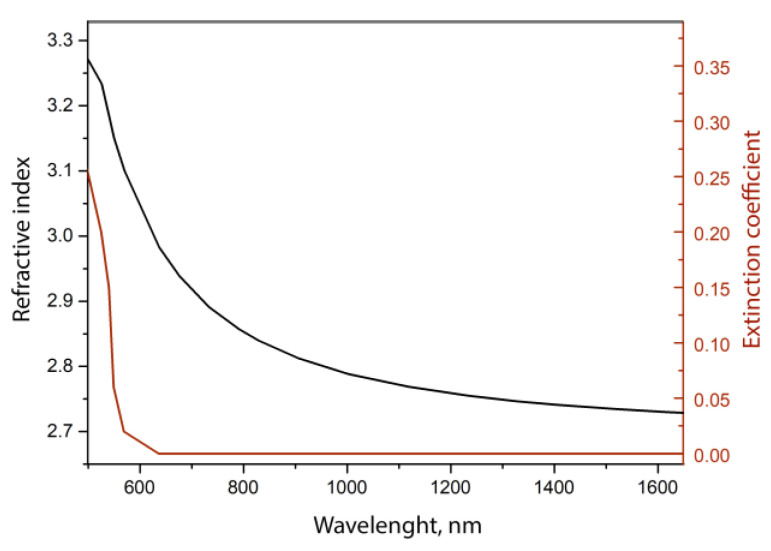
Calculated from ellipsometric measurements, the optical constants of a zinc telluride film with a thickness of 820 microns.

Previously, it was noted in [29,31,32,48] that the sensitivity of the lossy mode resonance to changes in the refractive index of the environment depends on the wavelength and refractive index of the coating, and it increases with an increase in any of these two parameters. Depending on the production method and conditions, the band gap of ZnTe films varies within 2.23–2.25 eV, but in any case, this material is optically transparent in the spectral range above 560 nm. The value of the real part of the complex refractive index of our films in the region of 600–1600 nm was determined using ellipsometric measurements. For this, ZnTe layers about 1 μm thick were deposited on plane-parallel quartz plates. The results of n and k calculations for one of the samples are shown in Figure 4. 

The value of the extinction coefficient in the range above 600 nm lies outside the domain of definition for a film of a given thickness, and, according to the model estimate, it is less than 0.01. From a comparison of the experimental values of the refractive index for the first (about 800 nm) and third (about 1550 nm) transparency windows of the telecommunications range, it can be seen that their difference is more than 0.1, which is a sufficiently large value for a significant change in the LMR sensitivity. One of the significant advantages of the visible spectrum sensor is that signal receivers are easy to build and have a low cost. In addition, such a comparison of sensitivities is only possible for multi-mode fibers, since a single-mode fiber in the visible region supports a multi-mode one.

**Figure 5:**
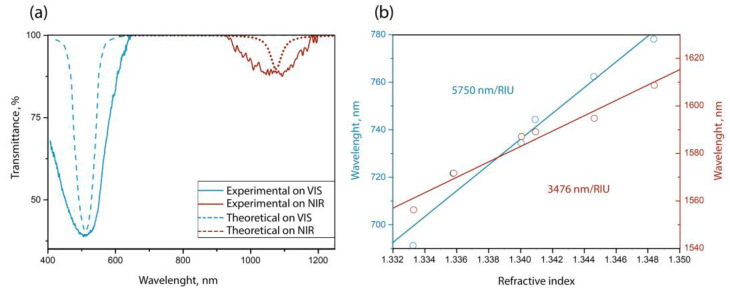
1st TM-LMRs in the visible (VIS) and near-infrared (NIR) spectral regions: (**a**) comparison of the transmission spectra obtained experimentally and calculated, (**b**) dependence of the resonance maxima on the refractive index of the studied NaCl solutions. Red and blue colors indicate data for sensors with a coating thickness of 96.3 nm and 35.8 nm, respectively.

Several sensors have been implemented with the LMR maximum located near 500 and 1100 nm in air, taking into account the subsequent shift of resonances in aqueous NaCl solutions with spectral operating ranges of 650–800 nm and 1550–1650 nm, respectively. Using the applied calculation method [25,49,50], we preliminary estimated the required thickness of the coatings to be grown. The calculation method is that the thinned section with the coating is represented as a Fabry–Perot interferometer. Since the light wave propagating in the fiber section is not polarized, the reflected power in s- and p-polarizations should be taken into account. The path of the light beam is a zigzag path due to multiple reflections at the fiber–coating interface. The main expression for transmission is [27,50,51]:

(3) $$P={\left[\frac{1}{2}{\left|\frac{r_{s1}+r_{s2}\exp\left(iϭ\right)}{1+r_{s1}r_{s2}\exp\left(iϭ\right)}\right|}^{2}+\frac{1}{2}{\left|\frac{r_{p1}+r_{p2}\exp\left(iϭ\right)}{1+r_{p1}r_{p2}\exp\left(iϭ\right)}\right|}^{2}\right]}^{M}$$

where $r_{s1}$ and $r_{p1}$ are the Fresnel coefficients for the s- and p-polarizations for the fiber–coating interface, and $r_{s2}$ and $r_{p2}$ are the Fresnel coefficients for the s- and p-polarizations for the coating–environment interface, respectively. $ϭ=-4\pi n_{1}dcos\theta_{2}\lambda^{-1}$ is the phase delay caused by the optical path difference, d is the thickness of the ZnTe coating, $\theta_{2}$ is the angle of incidence on the interface between the coating and the environment. M=L(rtanθ1) is the number of reflections at the fiber–coating interface, where L and r are the length and diameter of the thinned section, respectively, and $\theta_{1}$ is the angle of incidence on the fiber–coating interface. For the fundamental mode, $\theta_{1}$ can be expressed as θ1=arcsin⁡(βHE11(k0n2), where k0=2πλ is the fundamental mode propagation constant. Since one angle of incidence corresponds to one fixed wavelength for the fundamental mode, the interference dips transmissions at different wavelengths are presented as follows [52]:

(4) $$\lambda_{m}=\frac{4\pi n_{1}dcos\theta_{2}}{\varphi +2m\pi }$$

where *φ* is the phase delay caused by the Goos–Hänchen shift due to the total internal reflection at the coating–environment interface. Then, we obtain that, for a resonance near 500 nm, the coating thickness will be 35.8 nm, and for a position near 1100 nm, it will be 96.3 nm. Figure 5a shows the calculated and experimental transmission spectra of a multi-mode fiber sensor for given coating thicknesses when the environment is air.

It can be seen that the experimental dips are much wider than the calculated ones, and this is explained by the contribution of the conical parts of the thinned section to the transmission spectrum. Usually, flashing occurs only in the cylindrical segment of the thinned section, since the difference in the cones (insert in Figure 1) is sufficient for the fiber core to be removed from the coating at a distance at which the interaction is negligible. However, when working with a fiber in which the thickness is only 15 microns, any reduction in diameter will result in a strong interaction of light with the absorbing coating. Therefore, in the case of a multi-mode fiber, a wide dip in the transmission spectrum is caused by the emission over the entire surface of the thinned section of the optical fiber. 

Figure 5b shows the dependencies of the position of the resonance maximum for sensors with different operating wavelength ranges when filled with NaCl solutions of different concentrations. These concentrations of salt are quite easily soluble in water and do not form a precipitate in a short period. The sensitivity of a sensor optimized for the visible range is much higher than that of a similar device with an infrared operating range. In the visible range sensor, a higher sensitivity of 5750 nm/RIU has been achieved, and this is achieved by the fact that, in the visible region of the spectrum, the coating has a significantly higher refractive index.

**Figure 6:**
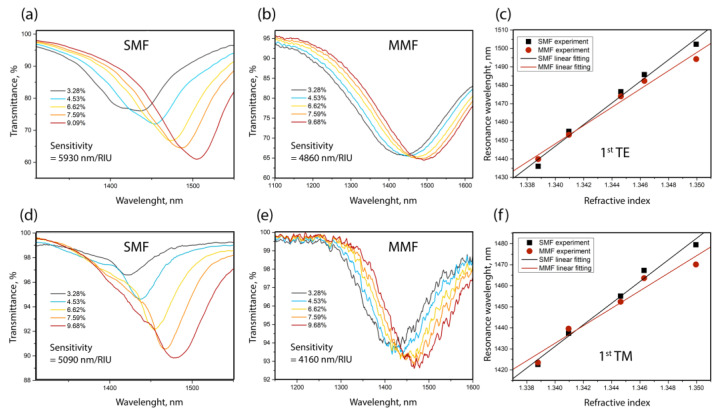
Transmission spectra of fiber sensors on SMF ((**a**)—TE, (**d**)—TM) and MMF ((**b**)—TE, (**e**)—TM) in aqueous solutions of common salt. The graphs indicate the mass percentage of salt in the solution. (**c**,**f**) Dependencies of the resonance maximum on the refractive index of the solution, connected by a linear approximation.

In order to correctly estimate the quality index, the value of which differs depending on the wavelength range [33,46,48], all coating thicknesses were implemented in such a way that the positions of the 1st TE- and TM-LMRs were near the same wavelength (~1450 nm). According to a theoretical estimate, this requires the thickness of the ZnTe coating for TE to be ~42.3 nm and that for TM to be ~127.5 nm. In addition, the half-width must also be estimated at an equal depth of resonances. If this condition was not met (for example, if the resonance depth was insufficient), the deposited coating was chemically removed from the multi-mode fiber, and its thinned sector was etched and re-deposited. In contrast to the approximately equal depths of TE and TM resonances in the multi-mode implementation of the sensor for the SMF, the depths differed significantly. In all our experiments, the diameter of the thinned section of a single-mode fiber was 26.3 µm, and for a multi-mode fiber, it varied and was equal to 108.6 and 99.5 µm for TE for TM modes, respectively. Changes in the transmission spectra of fiber sensors depending on the percentage of NaCl in the aqueous solution are shown in Figure 6.

It can be seen that the sensitivity of sensors based on a single-mode fiber is higher for both LMR components. We explain this fact by the fact that the surface roughness of the coating on a single-mode fiber is several times higher than that on a multi-mode fiber. Increased roughness leads to a larger area of contact of the coating with the analyzed medium and, consequently, to greater sensitivity. The processing data of AFM scans with the size 1 × 1 μm^2^ are shown in Table 1.

It can be seen from the table that the resulting surface roughness is affected by the final diameter of the etched fiber section. And this is despite the fact that the NH_4_F-(NH_4_)_2_SO_4_ etchant used in the work is considered polishing for silica. In our earlier work, we showed [53] that the rate of growth of a zinc telluride coating on a single-mode fiber increases with a decrease in the diameter of the thinned fiber section. The formation of thin films on the surface of substrates most often occurs in two stages: the nucleation stage, at which critical nuclei capable of further growth appear, and the film growth stage, at which critical nuclei grow and merge with each other to form a continuous film. On a rough or defective surface, specific growth mechanisms can operate that do not require the initial formation of nuclei. It is known that a less smooth surface contains a greater number of nucleation centers and thus can lead to a higher net deposition rate of the material. Based on the foregoing, it can be concluded that the surface treated with the etchant for a longer time has a greater roughness, which affects the acceleration of coating growth, in comparison with the untreated surface.

**Figure 7:**
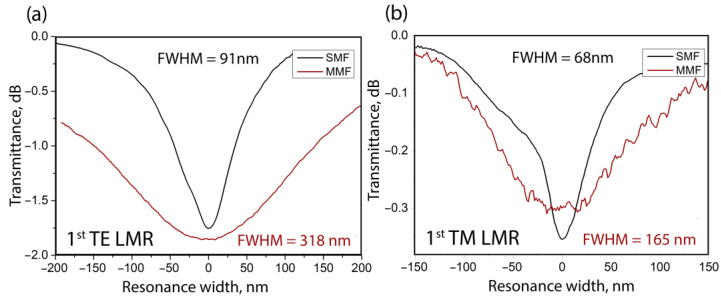
Shapes of resonances: (**a**) TE mode, (**b**) TM mode for sensors on SMF and MMF. The graphs show the Full Width at Half Maximum (FWHM) of the resonances.

Figure 7 shows a comparison of the shapes of the individual 1st LMR components, for sensors on single-mode and multi-mode fibers.

Resonances on the multi-mode fiber are wider, which, coupled with the lower sensitivity, gives a lower quality factor. Since the FOM is the ratio of the sensitivity to the resonance half-width, in the refractive index range of 1.33–1.35, it is 25 RIU^−1^ for multi-mode fibers and 75 RIU^−1^ for single-mode fibers.

## 4. Conclusions

In the study, a direct comparison of the influence of the type of optical fiber on the spectral characteristics and sensitivity of sensors for the mass concentration of aqueous solutions was demonstrated. The principle of operation of the sensors was based on the phenomenon of lossy mode resonance (LMR) obtained by applying a thin-film coating of zinc telluride (ZnTe) to a thinned segment of a silica fiber. During the MOCVD coating process, the transmission spectrum of the optical path was controlled in real time to monitor the position of the resonances and stop the synthesis process. The possibility of separating the first nine orders of resonances into electric (TE-) and magnetic (TM-) transverse components is demonstrated for the first time when working with a multi-mode optical fiber. The main parameters for comparing refractometers based on single-mode and multi-mode fibers were the sensitivity to changes in the refractive index of the surrounding media (SMRI), the spectral half-width (FWHM) of the resonance, as well as the operating wavelength range. A direct comparison of the characteristics of sensors with a working wavelength range in the visible (500–750 nm) and infrared (1350–1550 nm) ranges showed that the sensitivity in the first case is higher and amounted to 5750 nm/RIU. The difference in sensitivity for different measurement ranges is due to the dispersion of the refractive index of the zinc telluride coating. NaCl aqueous solutions with a refractive index range of 1.33–1.35 at a wavelength of 589 nm were used as test media for evaluating sensors. For the correctness of the comparison, all sensors on multi-mode and single-mode fibers were manufactured in such a way as to provide the same spectral range of operation and the depth of resonances. This was achieved by selecting the geometric parameters of the thinned section of the fiber on which the coating was applied, as well as by the ZnTe thickness. Comparing the shift of the 1st TM- and TE-LMRs with a change in SMRI determined that the maximum sensitivity obtained on the SMF fiber was 5930 nm/RIU, and on the MMF, it was about 4860 nm/RIU. The figure of merit of the sensors was 25 RIU^−1^ for the multi-mode fiber and 75 RIU^−1^ for the single-mode fiber. A higher sensitivity of sensors on SMF is achieved due to the higher (four times) roughness of the coating surface compared to implementation on MMF. The AFM data confirm the effect of the surface roughness of the etched silica glass on the optical quality of the ZnTe thin film. Due to the wide resonances driven by a large number of modes in a multi-mode fiber, the FOM is low compared to a single-mode sensor implementation, but the sensitivities differ not so much. At the same time, the mechanical strength, ease of operation at the stage of coating synthesis, and sufficiently high sensitivity of the final sensors allow us to conclude that multi-mode fiber is very promising for creating sensors in the visible range of the spectrum for determining the mass concentration of solutions. 

## Figures and Tables

**Table 1:** Comparison of sensitivity to the NaCl concentration of fiber refractometers based on the LMR effect.

Fiber Type	Etched Diameter (µm)	Etched Fiber		Coated Fiber	
		RMS (nm)	Roughness (nm)	RMS (nm)	Roughness (nm)
SMF	26.3	10.514	7.651	19.597	11.557
MMF	99.5	3.410	2.521	4.936	3.035

## Data Availability

Not applicable.
